# Leiomyosarcome de la vessie chez une patiente de 64 ans

**DOI:** 10.11604/pamj.2015.22.192.7681

**Published:** 2015-10-26

**Authors:** Mustapha Elkabous, Anwar Boukir, Asmaa Lakhdissi, Hamza Ettahiri, Fadila Kohen, Mohammed Afif, Youness Jabbour, Amal Drissy, Saber Boutayeb, Hassan Errihani

**Affiliations:** 1 Service d'Oncologie Médicale, Institut National d'Oncologie, Université Med V, Rabat, Maroc; 2 Service de Radiothérapie, Institut National d'oncologie, Université Med V, Rabat, Maroc; 3 Service d'Urologie B, CHU Ibn Sina, Université Med V, Rabat, Maroc; 4 Centre Anatomie Pathologique Hassan, Rabat, Maroc

**Keywords:** Sarcome de vessie, leiomyosarcome, tumeur de vessie, bladder sarcoma, leiomyosarcoma, bladder cancer

## Abstract

Le leiomyosarcome représente une tumeur rare de la vessie. Sa présentation clinique est non spécifique et dominée par l'hématurie. La résection endoscopique de la vessie avec un examen anathomopathologique permet de poser le diagnostic. La rareté de cette localisation ne permet pas d’établir une stratégie thérapeutique standard, néanmoins la chirurgie reste le traitement le plus utilisé. Nous rapportons le cas d'une patiente âgée de 64 ans, ayant présenté une hématurie. L'examen anatomopathologique d'une résection endoscopique de la vessie a posé le diagnostic d'un leiomyosarcome.

## Introduction

Les leiomyosarcomes vésicales sont des tumeurs rares. Seulement 5% des tumeurs de la vessie sont d'origine non épithéliale et moins de 0,5% sont des leiomyosarcomes, avec une centaine de cas rapportés dans la littérature [1, 2]. La rareté de cette localisation rend difficile l’établissement d'une stratégie thérapeutique standardisée. Malgré un traitement multimodal, le pronostic reste réservé [3]. Nous rapportons le cas d'une patiente âgée de 64 ans, présentant un leiomyosarcome de la vessie, découvert à la suite d'une hématurie macroscopique.

## Patient et observation

**Figure 1:**
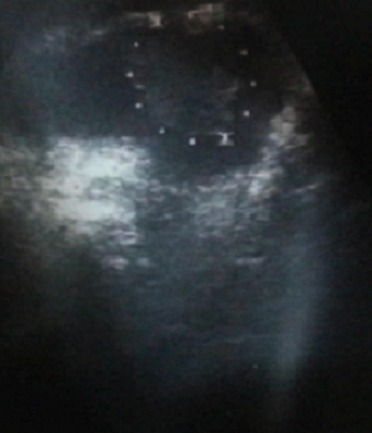
Echographie vésicale montrant une masse hétérogène au niveau de la face antérieure de la vessie

**Figure 2:**
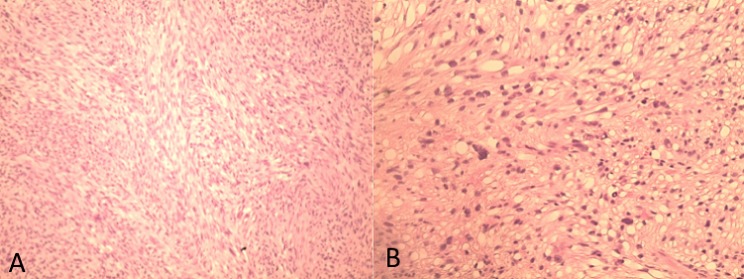
(a) coloration HES au grossissement 100 montrant une prolifération fusocellulaire en faisceaux entrecroisés; (b) Coloration HES au grossissement 200 montrant des atypies cellulaires marquees

**Figure 3:**
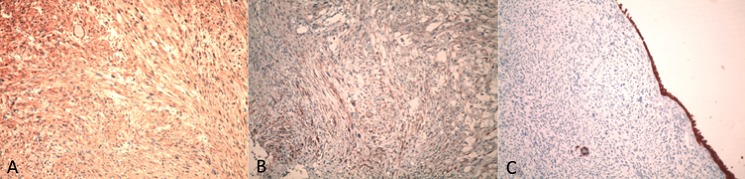
(a) marquage positif des cellules tumorales à l'anti-AML au grossissement 100; (b) marquage positif des cellules tumorales à l'anti-HCaldesmone au grossissement 100; (c) absence de marquage des cellules tumorales à l'anti-Cytokératine au grossissement 100 (clone AE1/AE3, DAKO) (exprimée par la muqueuse urothéliale)

**Figure 4:**
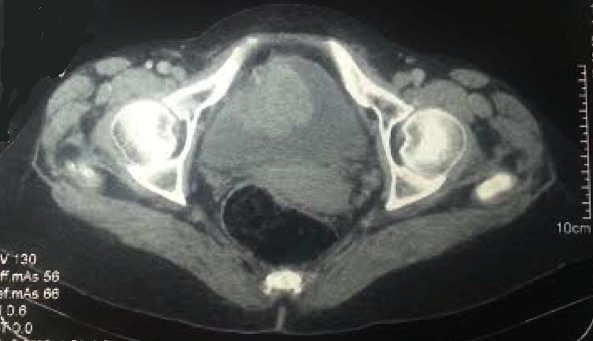
Image scannographique de la masse au niveau de la face antérieure de la vessie

Nous rapportons le cas d'une patiente âgée de 64 ans, sans notion de tabagisme, d'exposition aux nitrosamines ni aux hydrocarbures aromatiques polycycliques et sans antécédent de traitement par le cyclophosphamide ni par une radiothérapie pelvienne. Elle a présenté 4 mois avant le diagnostic une hématurie macroscopique, accompagnée d'une légère douleur pelvienne. L'examen clinique a trouvé une patiente avec une performance status à 0, asymptomatique avec un examen abdominal et des touchers pelviens sans particularité. Une échographie réalisée a mis en évidence une masse hypoéchogène hétérogène de la paroi antérieure de la vessie mesurant 50mm de grand axe (Figure 1). La patiente a bénéficiéd'une cystoscopie qui a visualisée une masse bourgeonnante au niveau de la paroi vésicale antérieure, saignant au contact. Une résection endoscopiquede la massea été réalisée permettant l'arrêt de l'hématurie et la diminution de la douleur pelvienne. L'examen anatomopathologique du matériel de résection a mis en évidence une prolifération tumorale maligne d'allure sarcomateuse de densité cellulaire élevée. Cette prolifération a été constituée de cellules parfois globoïdes ou souvent fusiformes, agencées en faisceaux entrecroisés, montrant une différentiation musculaire (Figure 2 a). Ces cellules étaient pourvues de noyaux arrondis ou ovalaires, irréguliers et nucléoles. Les atypies nucléaires étaientmarquées par la présence de cellules géantes tumorales multinucléolées (Figure 2 b). L'activité mitotique atteignait 15 mitoses par 10 champs au fort grossissement. La vascularisation était assurée par des vaisseaux parfois bordés directement par les cellules tumorales. Cette prolifération infiltrait la muqueuse urothéliale, qui était souvent ulcérée et remaniée par des foyers de nécrose, et le muscle: la tumeur est classée pT2. L'examen imunohistochimique avait mis en évidence un marquage positif et diffus aux anticorps anti-actine muscle lisse (AML) et anti-Hcaldesmone; le marquage à l'anticorps anti cytokératine était absent (Figure 3). Les résultats de l'examen anathomopathologique et immunohistochimique ont conclu à un leiomyosarcome de la vessie de haut grade. Un scanner thoraco-abdomino-pélvien a été réalisé, mettant en évidence un processus lésionnel de structure tissulaire, accolé à la paroi antérieure de la vessie, mesurant 50mm de grand axe (Figure 4). Aucune lésion secondaire à distance n'a été mise en évidence. La tumeur est donc classée T2N0M0. Une réunion de concertation pluridisciplinaire (RCP) a pris la décision de faire une cystectomie radicale avec mise en place d'une dérivation urinaire externe; décision que la patiente a accepté après explication du pronostic et du bénéfice de cette chirurgie.

## Discussion

Les tumeurs de la vessie représentent le 4^ème^ cancer par ordre de fréquence chez l'homme et le 12^ème^ chez la femme, dominées par les tumeurs urothéliales [4]. Les sarcomes vésicaux quant à eux sont rares, et ne représentent que 5% des tumeurs de la vessie, avec moins de 1% de leiomysarcome dans cette localisation [1, 2]. La symptomatologie clinique est dominée par les symptômes urinaires, principalement une hématurie macroscopique dans 81% des cas; mais le diagnostic est exceptionnellement suspecté à cette étape de l’évolution vu la rareté de cette pathologie [3, 5]. Plusieurs facteurs de risques ont étaient suggérés, principalement la mutation du gène du rétinoblastome, l'exposition au cyclophosphamide et la radiothérapie [2]. Les facteurs pronostiques décrits dans la littérature sont: la taille tumorale, la dédifférenciation, l'invasion lymphatique, les limites de résection, l'atteinte ganglionnaire et la présence de localisations secondaires [1, 3, 5]. La cystectomie (associée à une colpohysterectomie ainsi qu’à une résection de la collerette vaginale chez la femme) est le traitement de référence, malgré que la résection de la tumeur avec des marges saines suivie d'une radiothérapie n'est pas inférieure en termes de résultats au long terme. Des marges de résection de plus de 2 cm sont nécessaires pour un résultat cancérologique satisfaisant [3, 6, 7]. Une chimiothérapie néo adjuvante peut être proposée, et a permis, dans certains cas, une respectabilité ultérieure. L'intérêt de la chimiothérapie adjuvant n'est pas clairement défini. Cette dernière a été utilisée dans certaines séries et a permis d'améliorer la survie, Mais vu le petit nombre de cas dans les séries rapporter, ce bénéfice n'est pas significatif. La radiothérapie est recommandée en cas de marge de résection tumorale, mais a aussi été proposée dans certains cas après une chirurgie complète [5]. Malgré un traitement multimodal, le taux de récidive locale est de 16%, et de rechute à distance est de 51% (principalement au niveau pulmonaire, hépatique, osseux ou cérébral). La médiane de survie est de 46 mois, quant à la survie à 5 ans et à 10 ans est de 47% et 35% respectivement [3, 5].

## Conclusion

Les leiomysarcomes de la vessie sont des tumeurs rares. Ce diagnostic est rarement suspecté sur les examens cliniques et paracliniques. La prise en charge n'est pas standardisée, mais reste dominée par la chirurgie.
